# Modulation of performance, meat quality and gut health of broiler chicken in response to dietary nanoencapsulated lavender essential oil

**DOI:** 10.1038/s41598-025-23938-4

**Published:** 2025-11-14

**Authors:** Sheikh Adil, Muhammad T. Banday, Syed A. Hussain, Manzoor A. Wani, Azmat A. Khan, Islam U. Sheikh, Yasir Afzal, Zahid Kashoo, Showkat Shah, Sireen A.R. Shilbayeh, Gamal A. El-Shaboury

**Affiliations:** 1 Division of Livestock Production and Management, FVSc & AH, SKUAST-Kashmir, Shuhama, India; 2 Division of Livestock Production Technology, FVSc & AH, SKUAST-Kashmir, Shuhama, India; 3 Division of Animal Nutrition, FVSc & AH, SKUAST-Kashmir, Shuhama, India; 4 Division of Veterinary Microbiology and Immunology, FVSc & AH, SKUAST-Kashmir, Shuhama, India; 5 Division of Veterinary Pathology, FVSc & AH, SKUAST-Kashmir, Shuhama, India; 6 Department of Pharmacy Practice, College of Pharmacy, Princess Nourah bint Abdulrahman University, Riyadh, 11671 Saudi Arabia; 7 Biology Department, Faculty of Science, King Khalid University, Abha, 61413 Saudi Arabia

**Keywords:** Phytobiotics, Essential oil, Lavender, Nanoencapsulation, Broilers chicken, Systems biology, Zoology

## Abstract

The aim of this study was to evaluate the potential of nanoencapsulated lavender essential oil (LEO) as feed additive in broiler chicken. 420 male broiler chicks at one week of age were randomly distributed into 7 dietary treatments: CN (Control)-fed basal diet only; AB (Antibiotic)-basal diet + 10 mg/kg Enramycin; CS (Chitosan)-basal diet + 300 mg/kg chitosan nanoparticles; LEO_F200_ (basal diet + 200 mg/kg free LEO); LEO_F400_ (basal diet + 400 mg/kg free LEO); LEO_N200_ (basal diet + 200 mg/kg nanoencapsulated LEO) and LEO_N400_ (basal diet + 400 mg/kg nanoencapsulated LEO). Each group contained 4 replicates and each replicate had 15 birds fed with a corn-soybean based diet for 42 days of age. During the overall period (7–42 d), LEO_N200_ and LEO_N400_ exhibited significantly (*p* < 0.05) higher body weight gain and lowest feed conversion ratio than all other groups. Lowest (*p* < 0.05) feed intake was recorded in LEO_F400_ during all the periods (starter, finisher and overall). LEO_N400_ had higher (*p* < 0.05) dressing and cut-up part weights (*p* < 0.05) compared to other groups. Improved breast meat quality was recorded in LEO_N400_. Cecal coliforms decreased and lacotobacilli increased notably in LEO_N200_ and LEO_N400_ (*p* < 0.05). Duodenal and jejunal villus height increased (*p* < 0.05) in LEO_N200_ and LEO_N400_ groups. In conclusion, nanoencapsulated LEO at 200 and 400 mg/kg appears to be a potential antibiotic alternative for improving broiler chicken performance and gut health.

## Introduction

Antibiotic growth promoters (AGPs) have notably nurtured the poultry industry with improved feed efficiency and controlling infectious pathologies^1^. Use of AGPs began in 1940 s with the discovery of *Streptococcus aurefacians* in monogastric diet^2^. At subtherapeautic level, AGPs can be used in poultry production to promote growth and protect the health of birds by modifying the immune status mainly due to control of gastrointestinal infections and microbiota modification in the gut^3^. However, indiscriminate use of AGPs has led to the development of microbial resistance and also residues in the meat with serious implications for human and animal health and the environment. Many countries have enacted laws prohibiting use of AGPs in livestock production. Ban on AGPs and public health concerns have led researchers to look for alternatives, which are safe, natural and eco-friendly possessing various biological properties including antimicrobial, antioxidant and immune-modulation^4–6^. These include organic acids, enzymes, prebiotics, probiotics, postbiotics, phytogenics, aquatic plants etc^7–11^. Among these potential candidates, phytogenic feed additives have gathered attention in recent years as natural alternative antimicrobial agents^12^, improve growth and digestive functions^13^, modify luminal microbiota composition and gut integrity^14^.

Lavender (genus *Lavandula*), a phytogenic belonging to mint family (Lamiaceae), is extensively cultivated due to their rich content of biologically active compounds, particularly in the form of essential oils (EOs). Lavender essential oil (LEO) is one of the most often used essential oils in aromatherapy as it is said to possess antibacterial, antifungal, anti-inflammatory, and antiseptic qualities^15^. The main bioactive compounds include linalool, linalyl acetate and lavandulyl acetate^16^. Supplementation of LEO (@ 24 and 48 mg/kg diet as a feed additive, increased growth performance of broiler chicken was reported^17^. Adding broiler chicken diets with LEO improves growth performance mainly through ameliorating gut microbiota balance, intestinal structure, antioxidant capacity and also lowered blood cholesterol of broiler chickens^7^. EOs have been shown to possess antimicrobial effects, antifungal, antioxidant, antiviral, immunomodulatory and anti-inflammatory activities anticancer^5,18^.

The integration of various EOs as such into animal diets, however, has shown has demonstrated limitations because the majority of bioactive substances are volatile, hydrophobic, and readily degraded by light, air, and high temperatures^19^. Nanoencapsulation technology offers a promising approach to enhance bioavailability, improve utilization efficiency, enable controlled release, and precise targeting of these bioactive compounds. The controlled release of essential oils through encapsulation enhances the biochemical responses and promotes optimal nutrient absorption^20^. By permitting intracellular transit and prolonging their retention period within the cell, encapsulation of herbal essential oils has successfully concealed their disagreeable taste and aroma, preserved their antioxidant properties, and increased their therapeutic potential^21^. In vitro study has shown an enhancement in the antimicrobial potency of LEO by about three fold on encapsulation with hydroxypropyl-beta-cyclodextrin^22^. Encapsulation consists of a process in which the small particles are surrounded by a capsule coating. Encapsulating bioactive molecules at nanoscale (1–100 nm) is known as nanoencapsulation^23^. Because of its cationic charge, antibacterial potential, safety, biodegradability, and natural abundance, chitosan (CS) is thought to be the best biopolymer for nanoencapsulation^24^. The aim of this study was therefore to investigate the effect of nanoencapsulation of LEO as a green alternative to AGPs on the performance, meat quality and gut health of broiler chicken.

## Methods

### Ethics declarations

The current study was conducted in strict compliance with the regulatory framework established by the Committee for the Purpose of Control and Supervision of Experiments on Animals (CPCSEA), 2012, under the Prevention of Cruelty to Animals Act, 1960 of the Government of India. The Institutional Animal Ethics Committee (IAEC) at Faculty of Veterinary Sciences and Animal Husbandry, Sher-e-Kashmir University of Agricultural Sciences and Technology (SKUAST), Kashmir, reviewed and approved (SKUAST/IAEC-17/2023/110) all experimental protocols. Reporting of in Vivo Experiments (ARRIVE) criteria, which make sure that animal testing is done and reported in an ethical way.

## Material

The aerial parts of Lavender plant (*Lavandula officinalis*) grown at Field station, Council for Scientific and Industrial Research (CSIR) Bonera, Pulwama, J&K, India were collected and subjected to hydrodistillation in a Clevenger apparatus for extraction of essential oil. The extracted essential oil was then stored in refrigerator until use. The CS (derived from crab shell) was purchased from HiMedia laboratories Pvt. Ltd, India and Sodium triphosphate pentabasic (TPP) from Sigma-Aldrich. All other reagents used in the experiment were of analytical grade. All necessary permissions and approvals were obtained prior to the collection of plant material for this study and that the plant specimens were collected in compliance with institutional regulations and with the explicit consent of the relevant authorities.

## Analysis of LEO by gas chromatography-mass spectrometry

The presence of various bioactive compounds in LEO was analyzed using a gas chromatography-mass spectrometry (GC-MS) system (GC-MS/MS-7000D, Agilent Technologies, Palo Alto, CA, USA). The system was equipped with a splitless injector and a detector, both maintained at 280 °C. A 1 µL sample was injected onto an HP-5MS UI column (Agilent J&W, Santa Clara, CA, USA) measuring 30 m in length, 0.25 mm in internal diameter, and 0.25 μm in film thickness. Helium was used as the carrier gas at a constant flow rate of 1 mL/min. The oven temperature was programmed to range from 60 °C to 310 °C over a period of 40.5 min. Several bioactive chemicals found in LEO were identified by comparing their mass spectra and relative retention indices with those in the MS Library (Mass Spectral Library, NIST-17, Gaithersburg, MD, USA).

## Nanoencapsulation of LEO

**Fig. 1:**
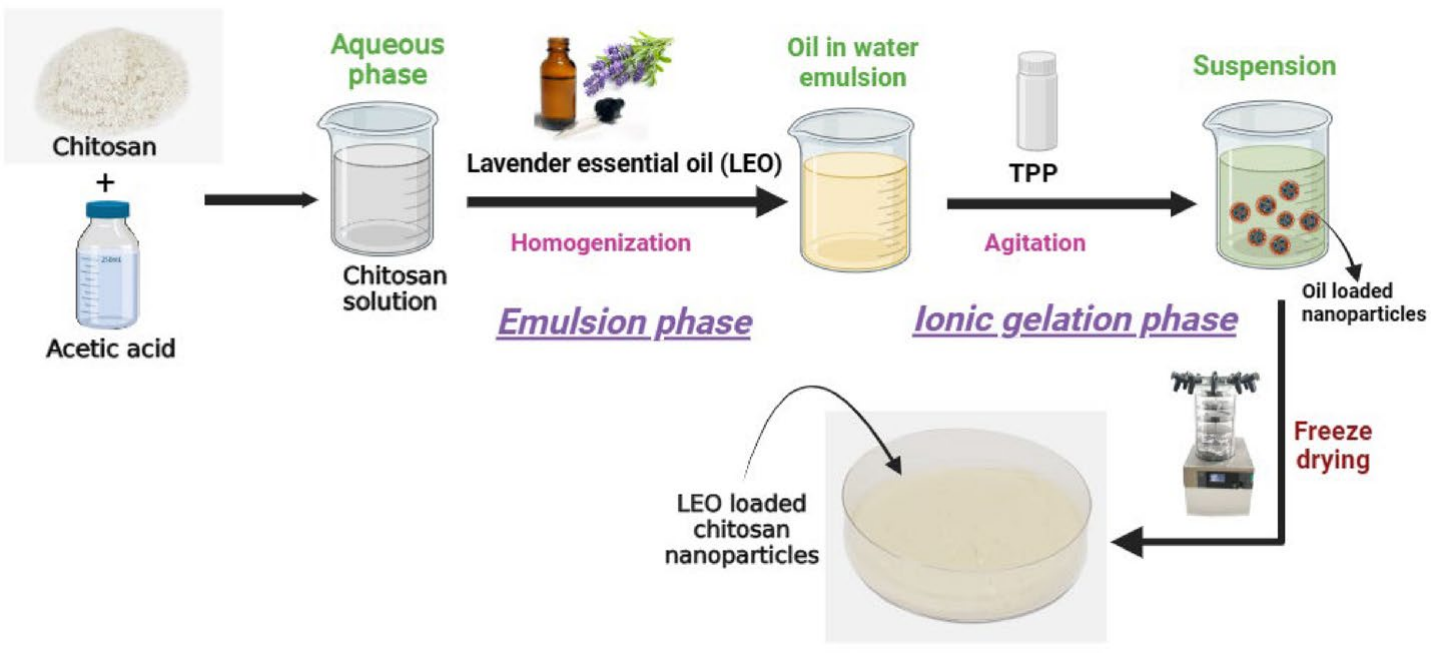
Nanoencapsulation of Lavender essential oil in chitosan through Ionic gelation method.

LEO was nanoencapsulated within chitosan nanoparticles (CNPs) using the ionic gelation method^25^, as illustrated in Fig. 1. After dissolving CS at a concentration of 1 mg/ml in 1% w/v acetic acid, the mixture was sonicated and centrifuged for 30 min at 10,000 rpm in a laboratory centrifuge (Eppendorf-5801R). To obtain a clear aqueous chitosan solution, the supernatant was collected and filtered through filter paper with a pore size of 1 μm. To create an oil-in-water emulsion, LEO was added drop by drop to this solution while it was being homogenized using a homogenizer (WiseTis^®^) in the appropriate ratio. The agitated emulsion was then subjected to a dropwise addition of a 1 mg/ml solution of TPP for approximately 40 min. After a second centrifugation at 10,000 rpm for 30 min at 4 °C, the solution was repeatedly washed with distilled water. It then underwent ultrasonication for 5 min at 40 kHz, followed by freezing at − 18 °C and subsequent freeze-drying using a freeze dryer (Lyovapor™ L-200, BUCHI). The aforementioned process was also used to prepare the unloaded CNPs without the addition of LEO. Before being used, all of the freeze-dried samples were kept at −18 °C in airtight containers.

## Morphological assessment of CNPs and LEO loaded CNPs

The morphological property of CNPs and LEO loaded CNPs was done by Scanning Electron Microscopy (SEM) technique using Hitachi 3600 N Scanning Electron Microscope having a 5 axis motorized stage coupled with ultra-dry compact EDS detector (Thermo scientific). The SEM images were obtained at 100KX magnification.

### Birds and experimental design

The experiment was conducted at Instructional Poultry Farm, Division of LPM, FVSc & AH, SKUAST-Kashmir, India. A total of 420 one-day old male Cobb broiler chicks (average body weight 42 ± 2.5 g) were procured from a local commercial hatchery and transferred to the Instructional farm. When the chicks were one week old, they were weighed, divided into 28 floor pens with sawdust as the litter, and raised for the duration of the 42-day trial. With seven treatments and four replications (15 birds per replicate), the experiment was carried out using a completely randomized design. Seven diets, mostly based on corn and soybeans, were created that were iso-caloric and iso-nitrogenous viz. CN (Control)-fed basal diet only; AB (Antibiotic)-basal diet + 10 mg/kg Enramycin; CS (Chitosan)-basal diet + 300 mg/kg chitosan nanoparticles; LEO_F200_ (basal diet + 200 mg/kg free LEO); LEO_F400_ (basal diet + 400 mg/kg free LEO); LEO_N200_ (basal diet + 200 mg/kg nanoencapsulated LEO) and LEO_N400_ (basal diet + 400 mg/kg nanoencapsulated LEO). Basal diet fed to broiler chicken is presented in Table 1. Feed and fresh water were provided *ad-libitum* during the whole experiment. The experiment was carried out in a completely sanitary environment, and the vaccination schedule and biosecurity precautions were closely adhered to. To give the birds a thermo-comfort environment, the initial temperature of the experiment was set at 95^0^F and then lowered by 5^0^F each week. For the first three days, the birds were given 24 h of light, after which the amount was reduced by 1 h each day until it reached 18 h, a pattern that was maintained until the conclusion of the experiment.

**Table 1:** Ingredients and nutrient composition of broiler chicken diets.

Ingredients (g/kg)	Starter (7–21 day)	Finisher (22–42 day)
Maize	550	586
Soybean meal	350	320
Fish meal	40	20
Vegetable oil	30	40
Limestone	7.00	10.00
Di-calcium phosphate	15.00	16.00
Salt	3.00	3.00
DL-Methionine	1.10	1.00
Lysine	1.30	1.40
Trcae mineral Premix^1^	1.00	1.00
Vitamin Premix^2^	1.50	1.50
Total	100.0	100.0
Nutrient composition		
Crude Protein*	223	203
Metabolizable Energy (Kcal/kg) **	3062	3150
Calcium*	10.10	10.50
Available P*	4.70	4.40
Lysine**	13.00	11.80
Methionine**	5.00	4.40

^1^Trace mineral premix (mg/kg diet): Mg 300, Mn 55, I 0.4, Fe 56, Zn 30 and Cu 4.

^2^Vitamin premix (per kg diet): Vitamin A 8250 IU, Vitamin D3 1200 ICU, Vitamin K 1 mg, Vitamin E 40 IU, Vitamin B1 2 mg, Vitamin B2 4 mg, Vitamin B1 210 mg, Niacin 60 mg, Pantothenic acid 10 mg, choline, 500 mg.

*Analyzed values **Calculated values.

## Sampling and data collection

### Growth performance

Every week, the cumulative feed intake (FI) for each replicate and the body weight (BW) of each individual bird were noted. For the starter (7–21 days), finisher (22–42 days), and whole duration (7–42 days) of the trial, body weight gain (BWG) and FI were calculated. For the same periods, the feed conversion ratio (FCR) was also calculated by dividing the FI by the cumulative BWG and correcting for any mortality.

### Carcass characteristics

On day 42, 2 birds per replicate (8 birds per treatment) were randomly chosen for sampling. After a 12 h fasting period, the sampled birds were individually weighed, then humanely euthanized under standardized conditions by Halal method of slaughtering. All procedures were conducted with strict adherence to animal welfare guidelines. Birds were handled gently to avoid stress, and the slaughter was performed swiftly by trained personnel using a sharp surgical-grade knife, ensuring immediate severance of the carotid arteries, jugular veins, trachea, and esophagus in a single stroke ensuring minimal distress to the birds. Following carcass processing, the dressed weight, weight of cut-up parts, and abdominal fat percentages were recorded from the sampled birds.

### Meat quality

The thigh meat samples were collected for assessing meat quality. The pH values of the samples were tested^26^ at 0 and 24 h following slaughter. Each of the 10 g portions of the homogenized samples was added with 100 ml of distilled water. Using a homogenizer, the process took one minute, and a digital pH meter (Tanco, India) was used to measure the pH levels.

In brief, 10 g of minced meat sample was combined with 15 ml of 0.6 M NaCl and left to stand at 4 °C for 15 min. After shaking the mixture and centrifuging it for 15 min at 5000 RPM, the supernatant fluid was poured off and water holding capacity (WHC) was calculated^27^.

The frozen breast meat samples were weighed as initial weight (W1). After the samples were weighed, they were sealed in zip-lock bags, labeled, and left to hang at 4 °C for a full day. After a second weigh-in, the final weight (W2) of the samples was noted. The Drip loss (DL) was calculated as:

$$\begin{aligned}\text{Drip loss}~(\%) = \frac{({\rm W}1 - {\rm W}2)}{{\rm W1}}\times 100\end{aligned}$$

Meat samples were analyzed for cholesterol levels according to the method described^28^. Cholesterol content in the extract was determined using a HITACHI UV-Spectrophotometer U-1800, set to a wavelength of 560 nm, after extracting 1 g of meat sample in 15 ml of a chloroform-methanol mixture (2:1 ratio).

The color parameters (L* for lightness, a* for redness, and b* for yellowness) of the breast sample cross-sections were measured using a Colorimeter (YS3060). Three measurements were taken at different times directly on the muscle surface of each sample, and the results were averaged.

Lipid peroxidation in the breast meat samples was evaluated by assessing thiobarbituric acid-reactive substances (TBARS), free fatty acids (FFA), and peroxide values at 0 and 5 days of storage. The determination of TBARS value was done as per standard procedure^29^. Additionally, a 5 g meat sample was mixed with 30 ml of chloroform for two minutes, with anhydrous sodium sulfate powder added. Filtration using No. 1 Whatman paper 40 into a conical flask came next^30^. The chloroform extract was titrated with 0.1 N alcoholic potassium hydroxide after two to three drops of 0.2% phenolphthalein indicator were added to get the pink color end point for the FFA value. Furthermore, to determine the peroxide value (PV), 30 ml of glacial acetic acid and 2 ml of potassium iodide solution were added to 25 ml of the chloroform extract. The mixture was left to stand for two minutes, with occasional shaking. It was then titrated with 0.1 N sodium thiosulphate until the endpoint was reached, which was marked by the non-aqueous layer turning white after adding 100 ml of distilled water and 2 ml of fresh 1% starch solution. The calculations were made as follows:

$$\begin{aligned} {\text{Free }} & {\text{fatty acid}}~(\% ) = \frac{{(0.1 \times {\text{vol}}{\text{. of KOH consumed}} \times 0.282)}}{{{\text{sample weight}}}} \times 100 \\ & {\text{Peroxide value}}~({\text{meq/kg}}) = \frac{{0.1 \times {\text{vol}}{\text{. of sodium thiosulphate consumed}}}}{{{\text{sample weight}}}} \times 100 \\ \end{aligned}$$

### Microbial examination

For microbiological analysis, the contents of the cecum were immediately collected into sterile polybags and stored at −20 °C. To achieve a 10^−1^ dilution, one gram of each sample was mixed with nine ml of sterile saline peptone solution and agitated for half an hour. In pre-sterilized tubes with 0.9 ml of sterilized normal saline solution and 0.1 ml of inoculum, serial 10-fold dilutions were prepared up to 10^−6^. For the growth of several microorganisms, readymade media from Hi-media Laboratories Pvt. Ltd., Mumbai, was utilized. A 0.1 ml aliquot of each dilution was plated onto various specialized media, including DeMan-Rogosa-Sharpe agar for lactic acid bacteria (LAB) (incubated at 37 °C for 48 h), MacConkey agar for coliforms (incubated at 37 °C for 24 h), and Plate Count Agar for total plate count (TPC) (incubated at 30 °C for 48 h). The TPC, coliform, and LAB counts in the cecal contents were determined^31^. The results were expressed as log10 colony-forming units per gram (cfu/g) after duplicate plates were prepared.

### Histomorphological study

Samples of the duodenum and jejunum, approximately 2 cm in length, were dissected using sterile scissors, washed with physiological saline to eliminate any contents, and then fixed in a 10% buffered formalin solution for later histomorphological examination. From the paraffin-embedded tissue blocks, sections (6 μm thick) were cut out, deparaffinized in xylene, and stained with hematoxylin and eosin^32^. Measurements were made using a microscope (Nikon Eclipse Ni) equipped with a digital camera (Nikon DS Ri2). The crypt depth (CD) and villus height (VH) were calculated by taking the mean of ten randomly selected portions from each sample. The VH to CD ratio was then computed^33^.

### Statistical analysis

Data were analysed using one-way ANOVA computer-aided statistical software package SPSS 20.0. The statistical model used was:

Y_ij_ = µ + T_i_ + e_ij_.

Where, Y_ij_ represents the observation for the dependent variables at the jth replicate in the ith treatment (i = 1 to 7), µ is the overall mean, T_i_ is the fixed effect of treatments (i = 1 to 7), and e_ij_ is the random error.

Duncan’s multiple range test was employed to compare the means. Individual data points were used as the experimental units for other parameters, while each replication served as the experimental unit for comparing performance. Data are presented as means ± SEM, and the significance was declared at *p* < 0.05.

## Results

### Gas chromatography-mass spectrometry

The results of gas chromatography of LEO are presented in Table 2. The results revealed the presence of major components in LEO as linalyl acetate (42.73%), linalool (27.14%), lavandulyl acetate (3.12%), Borneol (2.58%), Caryophyllene (2.26%), 1,8-Cineole (1.89%), E-β-Caryophyllene (1.77%), α-Terpineol (1.33%), Camphor (0.85%) and Octanone-3 (0.70%).

**Table 2:** Major bioactive compounds of lavender essential oil.

Bioactive compound	Retention index	Percentage
Octanone-3	983	0.70
1,8-Cineole	1030	1.89
Camphor	1147	0.85
Linalool	1154	27.14
Borneol	1159	2.58
α-Terpineol	1166	1.31
Linalyl acetate	1172	42.73
Lavandulyl acetate	1277	3.12
E-β-Caryophyllene	1425	1.77
Caryophyllene	1519	2.26

Identification by gas chromatography-mass spectrometry, National Institute of Standards and Technology (NIST, USA).

### Morphology of nanoencapsulated particles

**Fig. 2:**
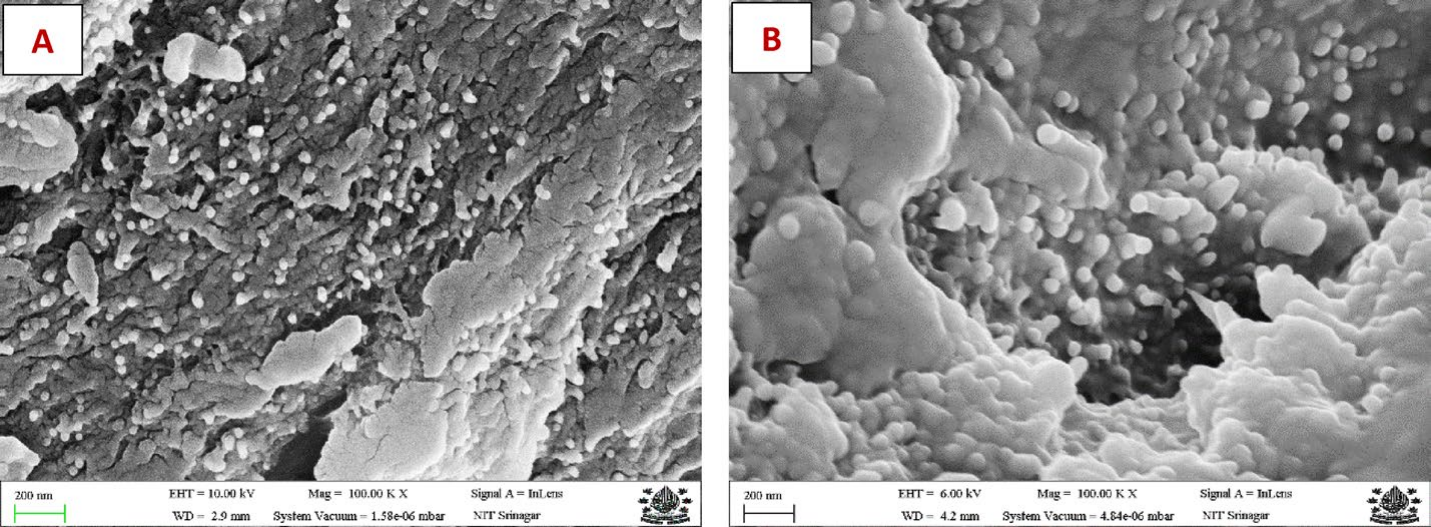
Scanning electron microscopy (SEM) of (**A**): Chitosan nanoparticles, (**B**): LEO loaded Chitosan nanoparticles.

The morphology of CNPs and LEO loaded CNPs analysed through scanning electron microscopy is presented in Fig. 2. The ionic gelation of CS with TPP resulted in spherical to irregular shaped nanoparticles. The surface of these nanoparticles showed grooves and ridges. However, the LEO loaded CNPs were spherical in shape, smooth surfaced and having aggregations in some locations. Also, the size of these particles showed an increase in size compared to unloaded CNPs. This could be due to encapsulation of LEO inside the CNPs.

### Growth performance

The results pertaining to growth performance of broiler chicks is presented in Table 3. During the starter (7–21 d) period, LEO_N400_ showed elevated (*p* < 0.05) BWG compared to all other groups including NC. BWG of CN, CS was comparable statistically. Likewise, AB, LEO_F200_ and LEO_N200_ showed no difference (*p* > 0.05) in the BWG among themselves. However, a decrease (*p* < 0.05) in the BWG was noticed in LEO_F400_ than all other groups. During the finisher (22–42 d) period and overall period (7–42 d), LEO_N200_ and LEO_N400_ exhibited higher (*p* < 0.05) BWG than all other groups. NC showed reduced BWG than other groups except LEO_F400_ that had even lowest (*p* < 0.05) BWG than NC. Lowest (*p* < 0.05) FI was recorded in LEO_F400_ during all the periods. Rest of the groups had no difference (*p* > 0.05) in the FI among themselves. During the starter (7–21 d) period, all the groups had similar (*p* > 0.05) FCR. Improved (*p* < 0.05) FCR was observed in LEO_N200_ and LEO_N400_ during overall period (7–42 d) than other groups.

**Table 3:** Effect of supplementing LEO (free and nanoencapsulated) on production performance of broiler chicken.

Parameter^1^	Dietary treatments^2^							SEM	*P*-value
	CN	AB	CS	LEO_F200_	LEO_F400_	LEO_N200_	LEO_N400_		
BWG, g									
7–21 d	528^b^	541^ab^	534^b^	536^ab^	457^c^	561^ab^	569^a^	7.44	0.003
22–42 d	1245^b^	1300^b^	1270^b^	1298^b^	1136^c^	1370^a^	1398^a^	16.75	< 0.001
7–42 d	1772^c^	1841^b^	1804^bc^	1834^b^	1593^d^	1931^a^	1967^a^	22.58	< 0.001
FI, g									
7–21 d	640^a^	640^a^	641^a^	632^a^	571^b^	638^a^	651^a^	6.50	0.006
22–42 d	2602^a^	2563^a^	2572^a^	2547^a^	2378^b^	2583a	2583a	16.75	0.014
7–42 d	3242^a^	3203^a^	3213^a^	3179^a^	2948^b^	3221^a^	3234^a^	20.12	0.027
FCR									
7–21 d	1.22	1.18	1.20	1.18	1.25	1.14	1.15	0.01	0.415
22–42 d	2.09^a^	1.97^bc^	2.03^ab^	1.96^bc^	2.09^a^	1.89^cd^	1.85^d^	0.02	< 0.001
7–42 d	1.83^ab^	1.74^c^	1.78^bc^	1.74^c^	1.85^a^	1.67^d^	1.65^d^	0.02	< 0.001

Means in a row with different superscripts differ significantly (*P* < 0.05).

^1^BWG, Body weight gain; d, day; FI, Feed intake; FCR, Feed conversion ratio.

^2^CN (Control)-fed basal diet only; AB (Antibiotic)-basal diet + 10 mg/kg Enramycin; CS (Chitosan)-basal diet + 300 mg/kg chitosan nanoparticles; LEO_F200_ (basal diet + 200 mg/kg free LEO); LEO_F400_ (basal diet + 400 mg/kg free LEO); LEO_N200_ (basal diet + 200 mg/kg nanoencapsulated LEO) and LEO_N400_ (basal diet + 400 mg/kg nanoencapsulated LEO).

### Carcass traits

The carcass traits of broiler chicken have been presented in Table 4. Pre-slaughter and dressed weights improved (*p* < 0.05) in LEO_N200_ and LEO_N400_ than NC. The weight of breast and thigh was higher (*p* < 0.05) in LEO_N200_ and LEO_N400_, however neck weight was comparable (*p* > 0.05) in all the dietary treatments and CN.

**Table 4:** Effect of supplementing LEO (free and nanoencapsulated) on carcass traits of broiler chicken.

Parameter^1^ (g)	Dietary treatments^2^							SEM	*P*-value
	CN	AB	CS	LEO_F200_	LEO_F400_	LEO_N200_	LEO_N400_		
PSLW	1926.52^e^	1998.11^c^	1957.00^de^	1989.27^cd^	1815.70^f^	2043.48^b^	2077.63^a^	18.06	< 0.001
Dressed weight	1361.66^e^	1425.25^c^	1385.95^d^	1425.31^c^	1276.62^f^	1473.35^b^	1500.26^a^	15.61	< 0.001
Breast weight	375.97^c^	412.22^b^	383.85^bc^	409.52^b^	328.65^d^	422.13^a^	443.32^a^	7.18	0.024
Thigh weight	185.17^cd^	209.59^abc^	191.65^bcd^	208.48^abc^	167.10^d^	218.85^ab^	225.87^a^	4.79	0.039
Neck weight	54.49	56.66	55.30	56.83	53.01	57.63	58.89	1.35	0.168

Means in a row with different superscripts differ significantly (*P* < 0.05).

^1^PSLW, Pre-slaughter live weight.

^2^CN (Control)-fed basal diet only; AB (Antibiotic)-basal diet + 10 mg/kg Enramycin; CS (Chitosan)-basal diet + 300 mg/kg chitosan nanoparticles; LEO_F200_ (basal diet + 200 mg/kg free LEO); LEO_F400_ (basal diet + 400 mg/kg free LEO); LEO_N200_ (basal diet + 200 mg/kg nanoencapsulated LEO) and LEO_N400_ (basal diet + 400 mg/kg nanoencapsulated LEO).

### Meat quality

The physical characteristics of thigh meat in broiler chicken are given in Table 5. pH value of thigh meat samples showed no statistical difference (*p* > 0.05) between CN and other treatments, however a numerical increase in pH was observed in free and nanoencapsulated LEO supplemented groups compared to CN at 15 min post-slaughter. The values of WHC, DL and Cholesterol revealed significant difference between CN and other groups. Better WHC was observed in all LEO supplemented groups with best values in LEO_N400_. A significant (*p* < 0.05) decrease in DL values was noticed in LEO_N200_ and LEO_N400_ in comparison to CN. The cholesterol content of thigh decreased (*p* < 0.05) in all LEO groups. The color coordinates (L, a and b) of thigh were comparable among all the groups and the values were within the normal range for a good quality meat.

**Table 5:** Effect of supplementing LEO (free and nanoencapsulated) on physical meat quality attributes of broiler chicken.

Parameter^1^		Dietary treatments^2^						SEM	*P*-value
	CN	AB	CS	LEO_F200_	LEO_F400_	LEO_N200_	LEO_N400_		
pH_15 min_	5.97	5.95	5.97	6.05	6.09	6.13	6.17	0.136	0.243
WHC (%)	48.31^c^	48.45^c^	48.04^c^	49.70^bc^	50.62^ab^	51.32^ab^	51.95^a^	0.335	< 0.001
DL (%)	3.42^a^	3.39^a^	3.41^a^	3.30^ab^	3.11^bc^	3.08^bc^	2.96^c^	0.044	0.004
Cholesterol (mg/kg)	87.67^a^	87.27^a^	87.12^a^	85.38^b^	83.71^c^	81.48^d^	80.10^d^	0.553	< 0.001
L*	54.70	55.31	55.83	56.00	54.96	55.34	55.75	0.341	0.762
a*	4.86	5.29	5.18	5.33	4.89	5.46	5.07	0.132	0.805
b*	7.21	7.56	7.54	6.93	7.28	7.45	7.82	0.209	0.570

Means in a row with different superscripts differ significantly (*P* < 0.05).

^1^WHC-water holding capacity; DL-drip loss; *L-lightness, a-redness, b- yellowness.

^2^CN (Control)-fed basal diet only; AB (Antibiotic)-basal diet + 10 mg/kg Enramycin; CS (Chitosan)-basal diet + 300 mg/kg chitosan nanoparticles; LEO_F200_ (basal diet + 200 mg/kg free LEO); LEO_F400_ (basal diet + 400 mg/kg free LEO); LEO_N200_ (basal diet + 200 mg/kg nanoencapsulated LEO) and LEO_N400_ (basal diet + 400 mg/kg nanoencapsulated LEO).

Furthermore, the results of lipid peroxidation parameters (TBARS, FFA and PV) of thigh meat in broiler chicken following supplementation of different levels of free and nanoencapsulated LEO have been presented in Table 6. These lipid peroxidation parameters at 0 and 5 days post-slaughter decreased (*p* < 0.05) on supplementation of LEO in the diet with greater reduction in LEO_N200_ and LEO_N400_.

**Table 6:** Effect of supplementing LEO (free and nanoencapsulated) on lipid peroxidation of thigh meat in broiler chicken.

Parameter^1^	Storage day		Dietary treatments^2^						SEM	*P*-value
		CN	AB	CS	LEO_F200_	LEO_F400_	LEO_N200_	LEO_N400_		
TBARS (mgMDA/kg)	0	0.86^a^	0.87^a^	0.84^a^	0.80^ab^	0.76^b^	0.69^c^	0.63^d^	0.023	0.012
	5	1.79^a^	1.81^a^	1.78^a^	1.67^b^	1.62^bc^	1.55^cd^	1.48^d^	0.021	< 0.001
FFA (%)	0	0.23^a^	0.22^a^	0.24^a^	0.20^ab^	0.19^ab^	0.15^b^	0.11^c^	0.013	< 0.001
	5	1.36^a^	1.34^a^	1.37^a^	1.26^b^	1.21^b^	1.13^c^	1.05^d^	0.022	0.035
PV (mEq/kg)	0	1.27^a^	1.30^a^	1.29^a^	1.19^ab^	1.12^bc^	1.02^cd^	0.93^d^	0.025	< 0.001
	5	3.03^a^	3.01^a^	3.04^a^	2.86^b^	2.70^c^	2.58^d^	2.41^e^	0.023	0.029

Means in a row with different superscripts differ significantly (*P* < 0.05).

^1^TBARS-thiobarbituric acid reactive substances; FFA-free fatty acids; PV-peroxide value.

^2^CN (Control)-fed basal diet only; AB (Antibiotic)-basal diet + 10 mg/kg Enramycin; CS (Chitosan)-basal diet + 300 mg/kg chitosan nanoparticles; LEO_F200_ (basal diet + 200 mg/kg free LEO); LEO_F400_ (basal diet + 400 mg/kg free LEO); LEO_N200_ (basal diet + 200 mg/kg nanoencapsulated LEO) and LEO_N400_ (basal diet + 400 mg/kg nanoencapsulated LEO).

### Microbial enumeration

Cecal microbial enumeration of broiler chicken at 42nd day of age is presented in Table 7. It was remarkably influenced (*p* < 0.05) in all the groups compared to CN. Cecal coliforms decreased (*p* < 0.05) and LAB increased (*p* < 0.05) notably in nanoencapsulated LEO groups (LEO_N200_ and LEO_N400_) compared to all other groups. Highest coliforms and lowest LAB were observed in CN. TPC decreased in the cecal contents of all treated birds compared to CN.

**Table 7:** Effect of supplementing LEO (free and nanoencapsulated) on cecal microbial population at 42 days post hatch in broiler chicken.

Parameter^1^ (Log_10_ CFU/g)	Dietary treatments^2^								SEM	*P*-value
	CN	AB	CS	LEO_F200_		LEO_F400_	LEO_N200_	LEO_N400_		
TPC	8.87^a^	8.54^c^	8.79^ab^		8.71^abc^	8.73^abc^	8.62^bc^	8.59^bc^	0.03	0.019
Coliform count	6.89^a^	6.48^bc^	6.73^ab^		6.55^bc^	6.51^bc^	6.32^c^	6.27^c^	0.05	0.004
LAB count	5.63^bc^	5.45^c^	5.57^bc^		5.77^ab^	5.79^ab^	5.89^a^	5.92^a^	0.04	0.005

Means in a row with different superscripts differ significantly (*P* < 0.05).

1CFU, Colony forming unit; TPC, Total plate count; LAB, Lactobacillus.

^2^CN (Control)-fed basal diet only; AB (Antibiotic)-basal diet + 10 mg/kg Enramycin; CS (Chitosan)-basal diet + 300 mg/kg chitosan nanoparticles; LEO_F200_ (basal diet + 200 mg/kg free LEO); LEO_F400_ (basal diet + 400 mg/kg free LEO); LEO_N200_ (basal diet + 200 mg/kg nanoencapsulated LEO) and LEO_N400_ (basal diet + 400 mg/kg nanoencapsulated LEO).

#### Duodenal and jejunal histomorphometry

**Fig. 3:**
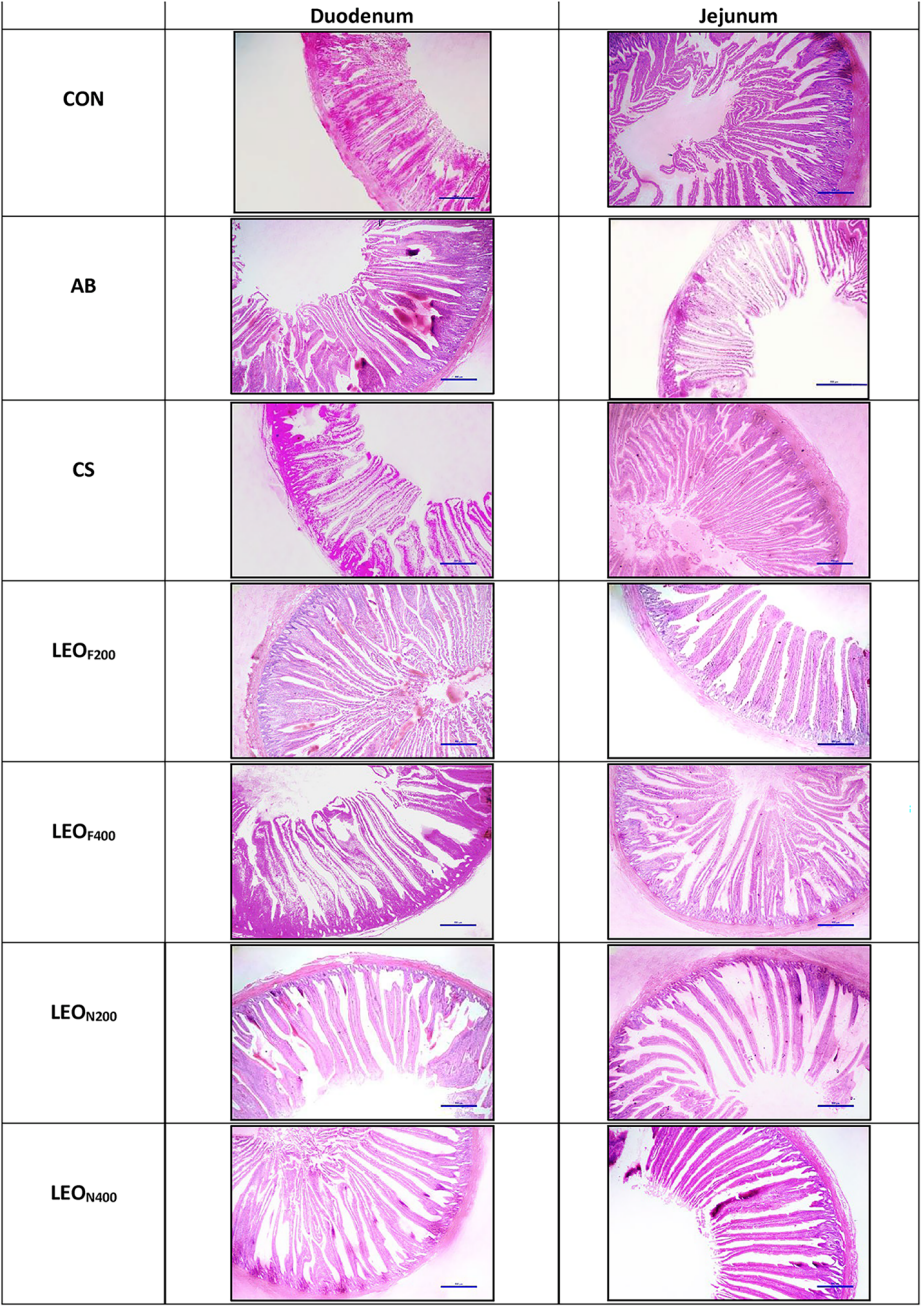
Representative histological micrographs of duodenum and jejunum in broiler chicken on day 42. Scale bar indicates 500 µm. 2CN (Control)-fed basal diet only; AB (Antibiotic)-basal diet + 10 mg/kg Enramycin; CS (Chitosan)-basal diet + 300 mg/kg chitosan nanoparticles; LEOF200 (basal diet + 200 mg/kg free LEO); LEOF400 (basal diet + 400 mg/kg free LEO); LEON200 (basal diet + 200mg/kg nanoencapsulated LEO) and LEON400 (basal diet + 400mg/kg nanoencapsulated LEO).

The histological micrographs and measurements of broiler chicken in various treatments on 42 day of age are shown in Fig. 3; Table 8. Both duodenal and jejunal VH increased significantly (*p* < 0.05) in LEO_N200_ and LEO_N400_ groups compared to CN. AB, LEO_F200_ and LEO_F400_ had comparable (*p* > 0.05) values for duodenal and jejunal VH, but improved than CN and CS. The CD values decreased remarkably in nanoencapsulated LEO groups (LEO_N200_ and LEO_N400_) than CN and other groups.

**Table 8:** Effect of supplementing LEO (free and nanoencapsulated) on the morphometry of duodenum and jejunum at 42 days post hatch in broiler chicken.

Parameter^1^	Dietary treatments^2^							SEM	*P*-value
	CN	AB	CS	LEO_F200_	LEO_F400_	LEO_N200_	LEO_N400_		
Duodenum (mm)									
VH	1465^d^	1519^bc^	1476^cd^	1536^b^	1533^b^	1583^a^	1589^a^	9.87	< 0.001
CD	226^a^	206^abc^	221^ab^	204^abc^	208^abc^	197^bc^	193^c^	3.25	0.047
VH: CD ratio	6.52^c^	7.38^abc^	6.71^bc^	7.57^ab^	7.45^ab^	8.04^a^	8.25^a^	0.15	0.002
Jejunum (mm)									
VH	1186^c^	1224^b^	1185^c^	1229^b^	1239^ab^	1269^a^	1267^a^	7.26	< 0.001
CD	212^a^	193^c^	208^ab^	192^a^	196^bc^	190^c^	188^c^	2.28	0.010
VH: CD ratio	5.62^b^	6.35^a^	5.71^b^	6.42^a^	6.34^a^	6.70^a^	6.78^a^	0.10	0.001

Means in a row with different superscripts differ significantly (*P* < 0.05).

^1^VH, Villus height; CD, Crypt depth.

^2^CN (Control)-fed basal diet only; AB (Antibiotic)-basal diet + 10 mg/kg Enramycin; CS (Chitosan)-basal diet + 300 mg/kg chitosan nanoparticles; LEO_F200_ (basal diet + 200 mg/kg free LEO); LEO_F400_ (basal diet + 400 mg/kg free LEO); LEO_N200_ (basal diet + 200 mg/kg nanoencapsulated LEO) and LEO_N400_ (basal diet + 400 mg/kg nanoencapsulated LEO).

## Discussion

In our study, the major 3 bioactive compounds in LEO recorded were linalyl acetate (42.73%), linalool (27.14%) and lavandulyl acetate (3.12%). These results corroborate with the earlier findings^16^ who reported the percentage of main 3 components in LEO as linalyl acetate (44.98%), linalool (25.27%) and lavandulyl acetate (3.44%).

The beneficial effects of EOs on growth performance of chicken are well established. EOs have been reported to stimulate the secretion of digestive enzymes^34^, reduce the incidence of intestinal diseases^35^, maintaining proper microbiota balance in the gut^5^. Additionally, EOs exert positive effects on BWG and FCR by exerting antioxidant, immunomodulatory effects, besides improving the histomorphology of intestines for better absorption of nutrients^5^. In our study, LEO_F200_ group showed better BWG and FCR than CN. Several other researchers have reported improved BWG and FCR in broiler chicken fed EOs in the diet. An increase in BWG in poultry birds with the supplementation of rosemary essential oil compared to control group has been reported^6^. In one more study LEO supplementation in the diet of broilers showed better BWG and FCR than control^17^. Earlier investigation has demonstrated that addition of LEO in the drinking water of broiler resulted in elevated BWG and decreased FCR^36^. Further, in our study nanoencapsulated LEO groups (LEO_N200_ and LEO_N400_) had best performance in terms of elevated BWG and lowest FCR than all other groups. This could be due to the fact that nanoencapsulation not only protects the bioactive compounds of EOs from degradation^37^ but also increases their penetration and effectiveness^38^. Nanoencapsulation enhances the stability and bioavailability of EOs, microbial stability and protects them against degradation^39^. BWG and FCR of broilers improved by supplementation of nanoencapsulated mint, thyme, and cinnamon EOs^40^. Furthermore, in our study LEO_F400_ group recorded reduction in the performance in terms of BWG and FCR than rest of the LEO groups (LEO_F200,_ LEO_*N*−200_ and LEO_*N*−400_). These results are in contrary to others^41^ documenting increased BWG and lower FCR in broilers supplemented with free LEO even up to 600 mg/kg. This inconsistency results may be due to plant genotype, physical and chemical soil conditions, harvest time, plant maturity and extraction process of EOs^42^. Interestingly in our study a reduction (*p* < 0.05) in the FI was observed in LEO_F400_, wherein free LEO was used @ 400 mg/kg than all other groups including CN. However, these results are in disagreement with other workers^43^ reporting no reduction in FI of broilers up to 600 and even 800 mg/kg LEO supplementation. The decrease in FI at higher levels of LEO supplementation in our study could be due to some bioactive compounds in LEO that might have intense smell and taste^44^, thereby resulting in decreased uptake by the birds. However, no such effect occurred in nanoencapsulated 400 mg/kg LEO group (LEO_N400_), depicting that the process of nanoencapsulation might have masked the strong smell and taste of those bioactive compounds responsible for reduction in FI.

In comparison to the control birds, dressed weights improved significantly in the birds supplemented with free (200 mg/kg) and nanoencapsulated LEO (200 and 400 mg/kg). These findings are consistent with previous findings^6^, who found a significant increase in the dressing % of birds provided nanoencapsulated rosemary essential oil in the feed. An increase in dressing percentage due to the addition of essential oils was reported earlier^45^. When compared to control, the weight of the breast and thighs improved dramatically, especially in the nanoencapsulated LEO groups. In comparison to control birds, broiler chickens supplemented with 75 and 150 mg/kg of turmeric essential oil showed a substantial increase in the relative weight of their breasts and thighs. These authors attributed the increased weight of important muscles to the potential impact of EOs on muscle synthesis. In contrary, no difference in the relative weight of the breasts and thighs after supplementation with lavender extract at 200, 300, or 400 ppm compared to control has been reported^46^.

There was a non-significant rise in the pH value of thigh meat in groups fed LEO in the diet, with more increase in LEO_N200_ and LEO_N400_, and the values were well within the range (5.9–6.2) for normal quality meat. A good quality meat has been reported to possess the pH range of 5.9 and 6.2^47^. In the current study, birds given nanoencapsulated LEO had a significantly higher WHC and a lower DL. A substantial increase in WHC and decrease in DL was reported by dietary essential oil supplementation when compared to the control^6,48^. The cholesterol level of thigh meat fell dramatically in birds given LEO, with the largest reduction shown in nanoencapsulated groups (LEO_N200_ and LEO_N400_). These findings are consistent with earlier study^6^ reporting that using nanoencapsulated rosemary EO in the feed resulted in a reduction in cholesterol levels in broiler chicken breast meat. The cholesterol-lowering impact of EOs in broiler chicken meat has been linked to the presence of numerous bioactive compounds that reduce HMG-CoA reductase protein production, resulting in a reduction in blood cholesterol levels^49^. The color of fresh chicken meat is thought to play an important role in customer meat preferences^50^. LEO supplementation, either free or nanoencapsulated, was found to maintain the colour coordinates of thigh meat in broiler chickens. Supplementation of free and nanoencapsulated EOs in broiler chicken had no deleterious influence on meat colour characteristics^6,51^.

Meat MDA concentration (TBARS), FFA, and PV were calculated to determine the level of meat lipid peroxidation. Poultry meat oxidizes more quickly than other meats due to its high concentration of unsaturated fatty acids^6^. According to the current study, the flesh of birds fed LEO showed a substantial decrease in TBARS, FFA, and PV, especially in nanoencapsulated groups. TBARS of thigh muscle were shown to be considerably reduced in birds treated with 300 and 400 ppm lavender extract^46^. FFA value and PV decreased in breast meat of broiler chicken fed thyme essential oil in the diet as compared to the control^52^. The presence of certain bioactive compounds in EOs that have the ability to scavenge radicals may be connected to the antioxidant capabilities^6^. Moreover, free radicals produced during the auto-oxidation process can be deactivated by EOs^53^.

The chicken’s microbial community is essential for immunity, disease resistance, and nutritional absorption^54^. The GIT contains both pathogenic (like coliforms) and beneficial (like LAB) bacteria. Pathogenic bacteria cause infection and toxin production whereas beneficial bacteria stimulate immune system and inhibit the growth of pathogenic bacteria^55^. The alteration of microbial community can affect the health and productivity of birds, help in detoxification and modulation of immune system^56^. Feed has an important role in modifying the microbial composition of intestines. EOs also modify intestinal microbiota in a positive way as recorded in the present study. A reduction in the number of coliforms and increment in the LAB count was observed in all the LEO supplemented groups with highly significant values in nanoencapsulated LEO groups (LEO_N200_ and LEO_N400_) compared to CN. These results are in agreement with the earlier reports documenting the similar trend of increased LAB counts and reduced coliforms with the dietary inclusion of free or nanoencapsulated EOs in broilers^57^.

The hydrophobicity of EOs has been linked to their antibacterial action via altering the permeability of cell membranes, which can result in cell death or leakage of contents^58^. EOs have been shown to negatively alter the coliform bacteria’s cell wall^59^. Other researchers also showed a decrease in coliforms when EOs were supplemented in the diet^5,60^. By generating lactic acid and bacteriocins, modifying the immune system, and competing with pathogenic bacteria for adhesion sites in the intestinal mucosa, LAB are crucial defenders against infection^61^. The improvement in the beneficial microbial community and reduction in the coliform counts could partly suggest the better performance of LEO supplemented groups reported in the present study.

The intestinal mucosa architecture reveals useful information about gut health of chicken. The villi of the small intestine play a crucial role in nutrient absorption. Shorter villi and deeper crypts have been linked to reduced nutrient absorption, decreased disease resistance, and lower performance^62^. A higher villus height (VH) may enhance digestive enzyme activity, thus improving digestibility^63^. In our study, dietary inclusion of LEO (free or nanoencapsulated) resulted in elevated VH in both duodenum and jejunum of broilers compared to CN. Highest improvement in VH was observed in nanoencapsulated LEO groups (LEO_N200_ and LEO_N400_). These results support the previous study^5^ reporting an increase in VH of duodenum and jejunum inbroilers fed diets containing EOs than control. Improvement in VH of broilers by supplementation of various EOs has also been demonstrated in earlier studies^57^. This improvement in VH has been related to the fact that EOs prevents villi damage by increasing antioxidant enzyme activity^64^. Some researchers^65^, however, ascribed the histological improvement to the decrease in toxins produced by EOs as a result of intestinal microbial alteration. In every group that received LEO supplementations, the CD decreased. The size of the crypt depth (CD) may reflect the rate of intestinal epithelial cell renewal, making it a key marker for assessing the health of chicken intestines^66^. Larger crypts suggest greater tissue turnover and an increased need for tissue replacement, which can indicate tissue injury^62^. Therefore, the reduced CD in our study’s LEO supplemented groups may indicate better gut health than CN. Further, there was an enhanced VH: CD ratio in LEO supplemented groups. Earlier findings^5^ also reported improved VH: CD ratio by providing EOs containing diet to broilers. A better VH: CD ratio has been associated with a favorable bacterial population in the intestines, particularly a higher count of lactic acid bacteria^67^. Therefore, the overall improvement in intestinal microstructure observed in our study may have enhanced digestive and absorptive functions, supporting the improved performance in the LEO groups compared to the CN group.

## Conclusion

The findings of this study clearly indicate that nanoencapsulated lavender essential oil supplemented at 200 mg/kg and 400 mg/kg diet considerably improved the overall performance and health condition of broilers. These groups outperformed other groups in terms of growth performance, as seen by increased body weight gain and decreased feed conversion ratios. Moreover, carcass characteristics and meat quality indices were positively influenced in these groups. Furthermore, birds in these groups had improved intestinal health, as demonstrated by better villus height in the duodenum and jejunum, as well as a healthier gut microbial balance. These enhancements indicate that nanoencapsulation not only improves the bioavailability and stability of lavender essential oil, but also increases its usefulness as a functional feed additive. Therefore, nanoencapsulated lavender essential oil at 200 and 400 mg/kg provides a viable natural alternative to antibiotic growth promoters in broiler chicken.

## Data Availability

The data presented in this study are available on request from the corresponding author.
